# Therapeutic Targeting of VEGFR-2, PD-L1, and EGFR–MET Pathways in Non-Small Cell Lung Cancer: Clinical Progress with Ramucirumab, Atezolizumab, and Amivantamab

**DOI:** 10.3390/jcm15083024

**Published:** 2026-04-15

**Authors:** Piotr Kawczak, Tomasz Bączek

**Affiliations:** 1 Department of Pharmaceutical Chemistry, Faculty of Pharmacy, Medical University of Gdańsk, 80-416 Gdańsk, Poland; tomasz.baczek@gumed.edu.pl; 2 Department of Nursing and Medical Rescue, Institute of Health Sciences, Pomeranian University in Słupsk, 76-200 Słupsk, Poland

**Keywords:** NSCLC, ramucirumab, atezolizumab, amivantamab, PD-L1, EGFR, VEGFR-2, MET pathway, targeted therapy, precision oncology

## Abstract

Non-small cell lung cancer (NSCLC) accounts for approximately 85% of all lung cancer cases and remains a leading cause of cancer-related mortality worldwide. Advances in molecular characterization and tumor biology have driven the development of antibody-based therapies targeting immune checkpoints, angiogenesis, and oncogenic signaling pathways critical for tumor growth and progression. Among these agents, Ramucirumab, Atezolizumab, and Amivantamab have demonstrated significant clinical efficacy in selected NSCLC populations. This review summarizes the mechanisms of action, pivotal clinical trials, and current clinical evidence supporting the use of ramucirumab, atezolizumab, and amivantamab in the management of advanced NSCLC. Relevant literature was identified through searches of PubMed, clinical trial registries, and recent international conference proceedings, with an emphasis on therapeutic efficacy, safety profiles, and rational combination strategies. Ramucirumab, a monoclonal antibody targeting vascular endothelial growth factor receptor-2 (VEGFR-2), has shown a survival benefit when combined with docetaxel in patients with previously treated advanced NSCLC. Atezolizumab, a programmed death-ligand 1 (PD-L1) immune checkpoint inhibitor (ICI), has become a cornerstone of NSCLC treatment across multiple disease stages, both as monotherapy and in combination with chemotherapy. Amivantamab, a bispecific antibody targeting both epidermal growth factor receptor (EGFR) and mesenchymal–epithelial transition factor (MET), has demonstrated robust and durable clinical activity in patients with EGFR exon 20 insertion–mutated NSCLC. Collectively, these agents highlight the expanding role of antibody-based therapies in NSCLC and underscore the importance of biomarker-driven patient selection and treatment personalization. Ongoing research into resistance mechanisms, predictive biomarkers, and combination approaches is expected to further refine the integration of antibody-based strategies in precision oncology for NSCLC.

## 1. Introduction

Lung cancer remains the leading cause of cancer-related mortality worldwide, accounting for approximately 1.8 million deaths annually, with NSCLC comprising nearly 85% of all diagnosed cases [1,2,3,4]. Despite advances in screening, imaging, and molecular diagnostics, the majority of patients continue to present with advanced or metastatic disease, for which long-term survival remains limited [5,6,7,8]. For decades, platinum-based chemotherapy served as the cornerstone of treatment for advanced NSCLC; however, its modest survival benefit and cumulative toxicity have driven the development of more precise and biologically informed therapeutic strategies [9,10,11,12,13,14]. Recent comprehensive updates further emphasize the shift toward personalized and biomarker-driven treatment paradigms, integrating genomic profiling and adaptive therapeutic sequencing into routine care [15,16].

Advances in molecular characterization and tumor immunobiology have fundamentally reshaped the therapeutic landscape of NSCLC, enabling the identification of oncogenic drivers, immune escape mechanisms, and dysregulated signaling pathways amenable to targeted intervention [17,18,19,20]. The integration of biomarker-driven therapies into routine clinical practice has translated into meaningful improvements in progression-free survival, overall survival, and quality of life for selected patient populations [21,22,23,24,25]. In particular, actionable alterations involving EGFR, ALK, ROS1, BRAF, MET, RET, and KRAS have become central to therapeutic decision-making, while emerging targets such as HER2 and B7-H3 are expanding the spectrum of druggable vulnerabilities [26,27]. While small-molecule tyrosine kinase inhibitors have been particularly effective in tumors harboring sensitizing driver mutations, monoclonal antibodies and antibody–drug conjugates have emerged as a complementary and increasingly important therapeutic class, targeting extracellular receptors, ligand–receptor interactions, and the tumor microenvironment [12,13,28,29]. Moreover, advances in tumor microenvironment research, resistance biology, and radiogenomic biomarkers are beginning to refine patient selection and therapeutic responsiveness [30,31,32].

Tumor angiogenesis represents a critical hallmark of NSCLC progression, with vascular endothelial growth factor (VEGF) signaling playing a central role in tumor growth, invasion, and metastatic dissemination [33,34,35,36]. Ramucirumab is a fully human monoclonal antibody that selectively binds vascular endothelial growth factor receptor 2 (VEGFR-2), thereby inhibiting ligand-induced receptor activation and downstream angiogenic signaling [37]. Clinical trials have demonstrated that ramucirumab, when combined with docetaxel, provides a statistically significant survival benefit in patients with previously treated advanced NSCLC, independent of histologic subtype [38,39,40]. These findings established ramucirumab as an effective antiangiogenic option in the second-line treatment setting and underscored the continued relevance of targeting tumor vasculature in advanced disease [41]. Ongoing research is exploring combination strategies integrating antiangiogenic agents with immunotherapy and targeted therapies to overcome resistance and enhance treatment efficacy [7,16].

In parallel with angiogenesis inhibition, immune checkpoint blockade has emerged as a transformative therapeutic strategy in NSCLC. PD-L1–mediated immune evasion represents a key mechanism by which tumors suppress anti-tumor immune responses [42,43]. Atezolizumab is a humanized IgG1 monoclonal antibody that targets PD-L1, preventing its interaction with programmed death-1 (PD-1) and B7.1 receptors and thereby restoring T-cell–mediated antitumor immunity [44]. Multiple phase II and III trials have demonstrated the clinical efficacy of atezolizumab across diverse NSCLC settings, including as monotherapy in previously treated disease, in combination with chemotherapy and antiangiogenic agents in the first-line metastatic setting, and as adjuvant therapy following surgical resection and platinum-based chemotherapy [45,46,47,48,49]. These studies have established atezolizumab as a cornerstone of modern NSCLC management and highlight the importance of immune modulation as a therapeutic pillar alongside targeted and cytotoxic approaches. Notably, resistance to immunotherapy—both primary and acquired—remains a significant challenge, driven by tumor-intrinsic factors, immune microenvironment heterogeneity, and adaptive signaling pathways [30,31].

More recently, the recognition of rare but clinically meaningful molecular subtypes has driven the development of innovative antibody-based strategies designed to overcome intrinsic and acquired resistance mechanisms. EGFR exon 20 insertion mutations represent a distinct subset of EGFR alterations associated with resistance to most first- and second-generation EGFR tyrosine kinase inhibitors and poor clinical outcomes [50,51,52]. Amivantamab is a fully human bispecific antibody targeting both EGFR and MET, engineered to inhibit ligand binding, promote receptor degradation, and engage immune effector mechanisms [53]. In the phase I CHRYSALIS trial, amivantamab demonstrated durable antitumor activity and a manageable safety profile in patients with platinum-pretreated NSCLC harboring EGFR exon 20 insertion mutations, leading to regulatory approval in this molecularly defined population [54,55]. Beyond EGFR exon 20 insertions, next-generation bispecific antibodies and antibody–drug conjugates—such as those targeting HER2 or B7-H3—are showing promising early clinical activity and may further expand treatment options for resistant disease [26,27].

To enhance conceptual clarity, this review adopts a mechanism-oriented framework to justify the integrated discussion of ramucirumab, atezolizumab, and amivantamab within the therapeutic landscape of advanced NSCLC. Although these agents are not unified by a single drug class, they represent three complementary antibody-based strategies that together exemplify modern precision oncology. Specifically, ramucirumab targets tumor angiogenesis through selective inhibition of VEGFR-2, thereby modulating the tumor vasculature; atezolizumab disrupts immune evasion via blockade of the PD-L1 axis, restoring antitumor immune responses; and amivantamab, as a bispecific antibody, simultaneously targets EGFR and MET signaling pathways, addressing oncogenic driver alterations and resistance mechanisms.

Taken together, these agents illustrate multi-level therapeutic intervention across key dimensions of tumor biology, encompassing vascular regulation, immune modulation, and direct inhibition of oncogenic signaling. Importantly, they also reflect distinct yet clinically relevant treatment contexts, including antiangiogenic therapy in the post-progression setting, immunotherapy across multiple lines of treatment, and targeted therapy in molecularly selected patient populations. Accordingly, this review is structured around a biologically integrated, precision oncology paradigm that emphasizes mechanistic diversity and clinical applicability, rather than grouping therapies solely by conventional drug class definitions. In aggregate, ramucirumab, atezolizumab, and amivantamab exemplify the expanding role of antibody-based targeted therapies in the contemporary management of NSCLC [4,8,15].

This narrative review provides a comprehensive overview of the mechanisms of action, key clinical trials, therapeutic positioning, and emerging combination strategies of ramucirumab, atezolizumab, and amivantamab, with a focus on precision oncology, biomarker-driven treatment selection, and evolving resistance mechanisms. A structured literature search of PubMed and Scopus identified English-language publications from 2006, selected to capture early translational and clinical investigations of targeted and immune-based therapies in NSCLC that preceded or accompanied regulatory approvals; the search was initially performed and subsequently updated during manuscript revision to include the most recent evidence through February 2026. Search terms included combinations of “NSCLC,” “ramucirumab,” “atezolizumab,” “amivantamab,” “EGFR,” “MET,” “VEGFR-2,” “ICIs,” “targeted therapy,” and “combination therapy.” Eligible studies comprised phase II and III prospective trials, randomized controlled trials, registration-directed studies, meta-analyses, and clinically informative prospective cohort studies. Priority was given to pivotal trials supporting regulatory approvals, while large real-world cohorts and retrospective analyses were included when they provided meaningful insights into treatment outcomes, safety, or sequencing strategies. Smaller translational or mechanistic studies were incorporated selectively to clarify mechanisms of action, resistance pathways, or biomarkers of response. Case reports, non-English publications, and preclinical-only studies were generally excluded unless they offered critical mechanistic insights relevant to the clinical activity or resistance patterns of the reviewed agents. Landmark studies predating 2006 were included when necessary to contextualize the biological rationale for targeting angiogenic signaling, immune checkpoints, or EGFR/MET pathways in NSCLC.

**Figure 1:**
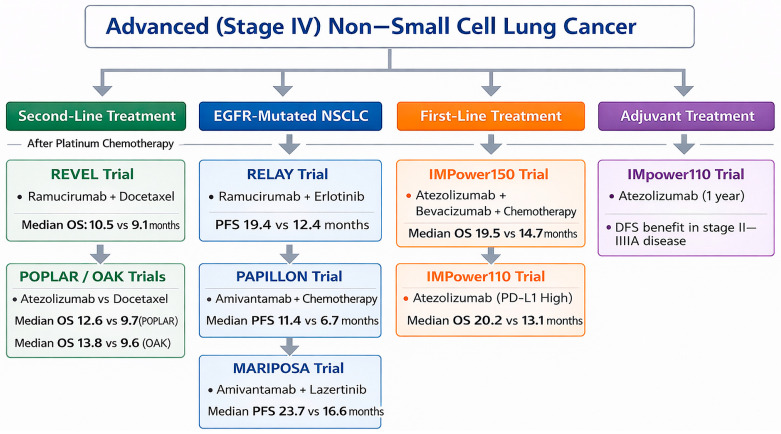
Schematic overview of treatment selection in advanced/stage IV non–small cell lung cancer, integrating pivotal clinical trial data by biomarker status and line of therapy. The scheme represents an original, interpretative synthesis of current evidence aligned with key NCCN and ESMO principles, including biomarker-driven treatment selection and therapy sequencing. It constitutes an original graphical representation and does not reproduce or replicate any specific published guideline or source. All abbreviations employed are defined in the text in the Abbreviations section.

Figure 1 presents a schematic representation of therapeutic decision-making in stage IV/advanced NSCLC, providing a structured, clinically oriented synthesis by integrating pivotal clinical trial data according to biomarker profile and line of treatment.

## 2. Ramucirumab

**Figure 2:**
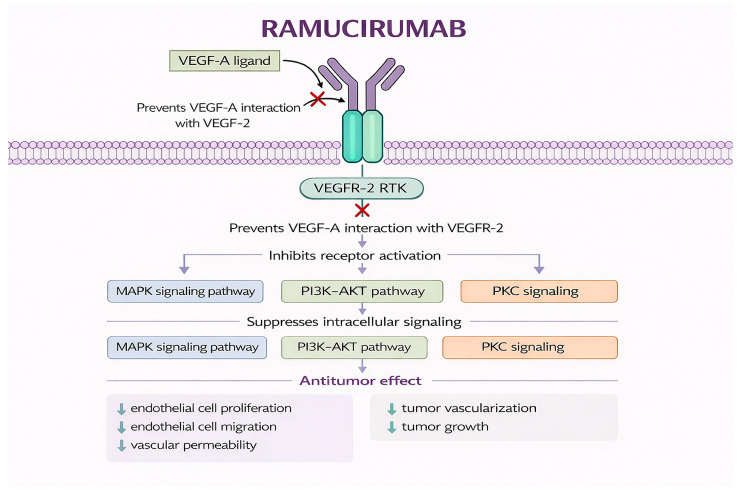
Mechanism of action of ramucirumab. Original schematic illustration created by the authors based on the mechanism described in [68]. Ramucirumab is a fully human monoclonal antibody that binds the extracellular domain of VEGFR-2 on endothelial cells, preventing VEGF-A interaction and subsequent receptor dimerization and autophosphorylation, thereby inhibiting downstream PI3K/AKT and PKC/RAS/RAF/MEK signaling pathways and ultimately reducing endothelial cell survival, proliferation, migration, progenitor cell mobilization, vascular permeability, and angiogenesis. All abbreviations employed are defined in the text in the Abbreviations section.

Ramucirumab is a fully human immunoglobulin G1 (IgG1) monoclonal antibody that selectively targets VEGFR-2, a central mediator of tumor angiogenesis in solid malignancies, including NSCLC [37,56,57,58]. By binding to the extracellular domain of VEGFR-2, ramucirumab blocks ligand-induced receptor activation by VEGF-A, VEGF-C, and VEGF-D, thereby inhibiting downstream signaling pathways involved in endothelial cell proliferation, migration, vascular permeability, and survival [59,60]. This receptor-specific mechanism distinguishes ramucirumab from other antiangiogenic agents such as bevacizumab, which neutralizes circulating VEGF ligands rather than directly inhibiting the receptor [61]. Given the pivotal role of angiogenesis in NSCLC progression, metastatic dissemination, and therapeutic resistance, VEGFR-2 inhibition represents a biologically sound therapeutic strategy [62,63,64,65,66,67]. Figure 2 shows its mechanism of action.

The clinical development of ramucirumab began with early-phase studies designed to evaluate its safety, pharmacokinetics, and pharmacodynamic activity across advanced solid tumors. A first-in-human phase I trial demonstrated acceptable tolerability, predictable pharmacokinetics, effective VEGFR-2 blockade, and preliminary antitumor activity, supporting further investigation in lung cancer populations [69]. These early studies confirmed that selective inhibition of VEGFR-2 could suppress angiogenic signaling without inducing excessive off-target toxicity, a limitation observed with earlier antiangiogenic approaches [70]. Based on these findings, ramucirumab advanced into later-phase trials across multiple malignancies, including gastric cancer, hepatocellular carcinoma, colorectal cancer, and NSCLC [71,72,73].

The pivotal evidence supporting the use of ramucirumab in NSCLC derives from several clinical trials evaluating its efficacy and safety across different therapeutic settings. The phase III REVEL trial demonstrated that the addition of ramucirumab to docetaxel significantly improved overall survival (OS), progression-free survival (PFS), and objective response rate compared with docetaxel plus placebo in patients with metastatic or stage IV NSCLC who had progressed after platinum-based chemotherapy [38]. Importantly, the trial included both squamous and non-squamous histologies, with consistent survival benefits observed across predefined subgroups, supporting the broad applicability of this regimen in previously treated disease [38,74]. Subsequent exploratory analyses confirmed efficacy regardless of prior exposure to EGFR tyrosine kinase inhibitors and in patients without identifiable oncogenic driver mutations [75,76]. However, interpretation of REVEL should consider potential confounding from subsequent therapies and the complexity of post-progression treatment patterns.

Combination strategies have been central to the clinical development of ramucirumab in NSCLC. The ramucirumab–docetaxel regimen remains the most extensively validated approach, with reproducible efficacy observed in both randomized trials and real-world settings [38,74,75,77]. Supporting evidence from the phase II JVBT study demonstrated clinical activity and acceptable tolerability in previously treated patients [78]. Additional studies, such as the phase II PLEURAM trial, have shown activity in specific clinical contexts, including patients with malignant pleural effusion following prior platinum-based chemotherapy [79,80]. However, these studies are limited by smaller sample sizes and, in some cases, a lack of comparator arms.

Ramucirumab has also been evaluated in biomarker-defined populations and earlier lines of therapy, particularly in EGFR-mutated NSCLC. The phase III RELAY trial demonstrated that ramucirumab combined with erlotinib significantly prolonged PFS compared with erlotinib alone in treatment-naïve patients with EGFR exon 19 deletion or L858R mutations [81,82,83]. Updated analyses, including RELAY+, have confirmed sustained overall survival benefit with dual EGFR–VEGFR inhibition [84]. Additional evidence from phase II trials such as RAMOSE suggests potential benefit when combined with third-generation EGFR inhibitors like osimertinib [85], supported by network meta-analyses indicating strong efficacy of EGFR–VEGF combination strategies in this setting [86]. However, these findings are derived from molecularly selected populations and may not be generalizable beyond these subgroups.

Emerging evidence also highlights the potential role of ramucirumab in challenging clinical scenarios, including central nervous system involvement and rare EGFR alterations. Studies such as RELAY-brain suggest activity in patients with brain metastases [87], while case reports describe responses in leptomeningeal disease and EGFR exon 20 insertion mutations [88,89]. Furthermore, the integration of circulating tumor DNA (ctDNA) to guide treatment decisions reflects the increasing importance of dynamic molecular monitoring in optimizing targeted therapy strategies [90].

More recently, combinations of antiangiogenic therapy with immune checkpoint inhibitors (ICIs) have gained attention. VEGFR-2 inhibition may modulate the tumor microenvironment by normalizing tumor vasculature, enhancing immune cell infiltration, and reducing immunosuppressive signaling, thereby augmenting immunotherapy efficacy [91,92]. Clinical studies, including combinations such as pembrolizumab plus ramucirumab, have demonstrated promising activity [93]. Additionally, a phase II trial evaluating docetaxel, ramucirumab, and pembrolizumab showed encouraging antitumor activity in previously treated NSCLC [94], while the Lung-MAP S1800A study demonstrated improved overall survival with ramucirumab plus docetaxel compared with standard-of-care therapy following prior immunotherapy [95]. Early-phase studies combining ramucirumab with PD-1/PD-L1 inhibitors have also reported promising results, although definitive phase III data remain pending [96].

The safety profile of ramucirumab is consistent with its antiangiogenic mechanism. Common adverse events include hypertension, fatigue, neutropenia, proteinuria, and bleeding, most of which are manageable with supportive care and dose modification [38,97]. In the REVEL trial, higher rates of grade ≥ 3 adverse events were observed, largely attributable to docetaxel-associated toxicity [38]. Importantly, severe pulmonary hemorrhage was not significantly increased, supporting its safety across histologic subtypes [98].

Despite its established clinical benefit, a critical limitation in the clinical implementation of antiangiogenic therapies in NSCLC is the persistent absence of validated predictive biomarkers for VEGFR-2 inhibition. Exploratory analyses have investigated circulating VEGF levels, tumor VEGFR expression, and angiogenic gene signatures, but none have demonstrated sufficient reproducibility or predictive value for routine clinical use [99,100]. This “biomarker gap” reflects several interrelated biological and methodological challenges. Tumor heterogeneity—both spatial and temporal—substantially complicates biomarker identification, as angiogenic signaling varies across tumor regions and evolves during disease progression. Angiogenic redundancy further limits biomarker utility, as compensatory activation of alternative pathways such as FGF, PDGF, and ANGPT/TIE2 can bypass VEGFR-2 blockade. In addition, angiogenesis is dynamically regulated by the tumor microenvironment, including hypoxia and stromal interactions, which influence vascular remodeling in a context-dependent manner. Finally, candidate circulating and tissue-based biomarkers—including VEGF ligand levels, soluble receptors, and microvessel density—have shown inconsistent and non-reproducible associations with clinical outcomes across trials. These factors explain why patient selection for ramucirumab remains largely based on clinical characteristics rather than biomarker-driven stratification, in contrast to EGFR- and MET-targeted approaches. This represents a key unmet need in the optimization of antiangiogenic therapy and underscores the importance of ongoing research aimed at identifying robust angiogenesis-related biomarkers and refining biomarker-driven treatment algorithms, particularly in combination with immunotherapy [99,100,101].

Beyond NSCLC, ramucirumab has demonstrated clinical efficacy in other malignancies, particularly gastric and gastroesophageal junction cancers, reinforcing the broader relevance of VEGFR-2 inhibition [102]. As the NSCLC treatment landscape continues to evolve, ramucirumab remains an important option in later-line therapy, particularly for patients who progress after platinum-based chemotherapy and immunotherapy and lack actionable molecular alterations [67,103].

Table 1 summarizes the treatment-emergent adverse events associated with ramucirumab together with recommended management strategies, whereas Table 2 presents the key pivotal clinical trials evaluating this agent in NSCLC.

## 3. Atezolizumab

**Figure 3:**
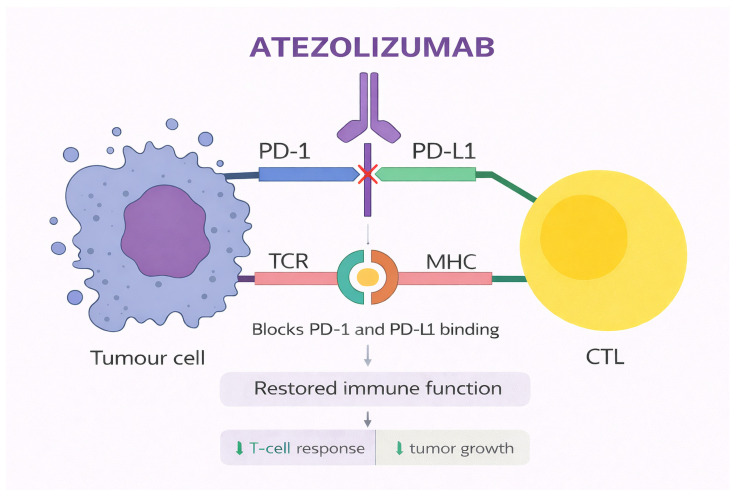
Mechanism of action of atezolizumab. Original schematic illustration created by the authors based on the mechanism described in [112]. Cytotoxic T lymphocytes recognize tumor-associated antigens presented by MHC on tumor cells via the TCR, but interaction of PD-1 on CTLs with PD-L1 on tumor cells suppresses this response, whereas atezolizumab, a monoclonal antibody targeting PD-L1, blocks this interaction, thereby restoring T-cell activation, enhancing antitumor immunity, and promoting tumor cell killing. All abbreviations employed are defined in the text in the Abbreviations section.

Atezolizumab is a humanized IgG1 monoclonal antibody directed against PD-L1 and represents one of the most extensively studied ICI in NSCLC [44]. By binding PD-L1 expressed on tumor cells as well as antigen-presenting cells within the tumor microenvironment, atezolizumab prevents PD-L1 interactions with programmed death-1 (PD-1) and the costimulatory molecule B7.1 (CD80), thereby restoring T-cell activation, proliferation, and effector function [105]. Unlike antibodies targeting PD-1 directly, atezolizumab preserves PD-L2–PD-1 signaling, which may contribute to differences in immune homeostasis and potentially influence toxicity patterns across ICIs [106]. In addition, atezolizumab is engineered with a modified Fc region that minimizes binding to Fcγ receptors, thereby limiting antibody-dependent cellular cytotoxicity against activated T lymphocytes while maintaining effective immune checkpoint blockade [107]. These structural and functional features provide the biological foundation for its activity and tolerability in NSCLC. Figure 3 depicts the mechanism of action of the agent.

The clinical development of atezolizumab began with the first-in-human Phase I PCD4989g study (MPDL3280A), which evaluated safety, pharmacokinetics, pharmacodynamics, and preliminary antitumor activity across multiple advanced solid tumors, including NSCLC [108]. This early-phase trial demonstrated durable objective responses and prolonged disease control in a subset of patients, even among those who had received multiple prior lines of therapy, while establishing a manageable safety profile [109]. These findings supported continued development in lung cancer–specific cohorts and highlighted PD-L1 expression as a potential predictive biomarker. Subsequent Phase II trials, including the BIRCH and FIR studies, further explored atezolizumab monotherapy in PD-L1–selected advanced NSCLC populations and confirmed clinically meaningful antitumor activity, particularly in patients with high PD-L1 expression on tumor cells or tumor-infiltrating immune cells [110,111]. However, these early-phase and biomarker-enriched studies should be interpreted with caution given their non-randomized design and potential selection bias, which may overestimate treatment effects.

Atezolizumab has been extensively evaluated across multiple therapeutic settings in non–small cell lung cancer (NSCLC), including previously treated disease, first-line metastatic therapy, and earlier-stage adjuvant treatment. Early clinical evidence was provided by the randomized phase II POPLAR trial, which demonstrated improved overall survival (OS) with atezolizumab compared with docetaxel in patients with previously treated advanced or metastatic NSCLC following platinum-based chemotherapy (median OS 12.6 vs. 9.7 months; HR 0.73) [113]. These findings were subsequently confirmed in the pivotal phase III OAK trial, which showed a statistically significant and clinically meaningful OS benefit for atezolizumab over docetaxel in the intention-to-treat population (13.8 vs. 9.6 months; HR 0.73) across both squamous and nonsquamous histologies [44,114,115]. Importantly, clinical benefit was observed across all PD-L1 expression subgroups, although the magnitude of benefit was greater in PD-L1–positive tumors. Extended follow-up analyses also identified a subset of long-term survivors, supporting the potential for durable disease control with immune checkpoint blockade [116]. Additional biomarker analyses from these trials suggest that molecular features such as TP53 status may further refine response prediction [117]. On the basis of these results, atezolizumab received regulatory approval for the treatment of metastatic NSCLC after progression on platinum-based chemotherapy, including after appropriate targeted therapy in patients with EGFR or ALK alterations [118]. Nevertheless, interpretation of OAK and POPLAR should consider their open-label design, which may introduce bias in outcome assessment, and the potential impact of subsequent therapies and crossover on overall survival estimates.

Following success in the second-line setting, atezolizumab was investigated as first-line therapy in metastatic NSCLC. The phase III IMpower110 trial demonstrated that atezolizumab monotherapy significantly improved OS compared with platinum-based chemotherapy in patients with PD-L1–high EGFR/ALK wild-type metastatic NSCLC (20.2 vs. 13.1 months; HR 0.59) [119]. These findings established atezolizumab monotherapy as an effective first-line option in biomarker-selected patients and underscored the importance of molecular and immunologic profiling in treatment selection. For patients ineligible for platinum-based chemotherapy, the phase III IPSOS trial demonstrated that atezolizumab monotherapy significantly improved survival compared with single-agent chemotherapy, supporting its role in frail or vulnerable populations [120,121,122]. However, these results are largely restricted to biomarker-defined or clinically selected populations, which may limit generalizability to broader patient groups. Because a substantial proportion of patients present with low or negative PD-L1 expression, several combination strategies were developed to broaden the clinical applicability of atezolizumab-based therapy.

The phase III IMpower150 trial evaluated a quadruplet regimen of atezolizumab, bevacizumab, carboplatin, and paclitaxel in metastatic nonsquamous NSCLC and demonstrated significant improvements in both OS and progression-free survival (PFS) compared with bevacizumab plus chemotherapy alone [45,123]. This regimen highlighted the potential synergy between immune checkpoint inhibition and angiogenesis blockade. Final and exploratory subgroup analyses confirmed benefit even in clinically challenging populations, including patients with EGFR mutations and brain or liver metastases [124]. More recent phase III evidence from IMpower151 further supports the efficacy of atezolizumab-based chemo-immunotherapy and antiangiogenic combinations in metastatic nonsquamous NSCLC [125]. Additional phase III studies further supported the integration of atezolizumab into platinum-based chemotherapy regimens. The IMpower130 trial demonstrated improved OS and PFS with atezolizumab plus carboplatin and nab-paclitaxel in previously untreated metastatic nonsquamous NSCLC [46]. In patients with metastatic squamous NSCLC, the IMpower131 trial showed a significant improvement in PFS with atezolizumab combined with chemotherapy, although the OS difference did not reach statistical significance [126]. The IMpower132 trial further confirmed the safety and efficacy of atezolizumab combined with platinum and pemetrexed [127,128]. Notably, heterogeneity in trial design, chemotherapy backbones, and patient populations across the IMpower program complicates cross-trial comparisons and may contribute to variability in observed outcomes.

Beyond metastatic disease, the therapeutic role of atezolizumab has expanded into earlier-stage NSCLC. The phase III IMpower010 trial demonstrated that adjuvant atezolizumab after surgery and chemotherapy significantly improved disease-free survival, particularly in PD-L1–positive stage II–IIIA disease [47,129]. Long-term follow-up, including 5-year outcomes, supports the durability of benefit in this setting [130], while real-world registry data further validate effectiveness in routine clinical practice [131]. In locally advanced disease, emerging phase II data suggest that integrating atezolizumab into multimodal strategies including chemo-radiotherapy may further improve outcomes [132]. Taken together, these findings represent a major shift toward incorporation of immunotherapy into curative-intent treatment paradigms [133,134], although longer follow-up and mature overall survival data remain necessary to fully define long-term benefit in earlier-stage disease.

The safety profile of atezolizumab is consistent with immune checkpoint inhibition and is characterized by immune-related adverse events resulting from nonspecific immune activation. Common toxicities include fatigue, rash, pruritus, diarrhea, and endocrinopathies, while less frequent but clinically significant events include immune-mediated pneumonitis, hepatitis, colitis, nephritis, and neurologic toxicities [135]. Recent meta-analyses have further characterized the risk of pneumonitis across ICIs, confirming its clinical relevance in lung cancer populations [136]. Additional real-world and translational studies highlight the importance of immune monitoring, including peripheral T-cell profiling and proteomic signatures, in understanding toxicity and response variability [137,138]. Emerging data indicate that elevated anti-atezolizumab antibodies may influence pharmacokinetics and clinical outcomes, underscoring the need for further investigation of treatment immunogenicity [138].

Immune-mediated pneumonitis is a particularly important concern in patients with NSCLC due to underlying lung disease, prior thoracic radiotherapy, and its overlapping clinical and radiographic features with infection or disease progression, while management of immune-related adverse events follows established guidelines that emphasize early recognition, treatment interruption, corticosteroid administration, and escalation to additional immunosuppressive therapies when required [139,140]. Novel observational approaches, including digital phenotyping methods such as tongue imaging, have also been explored as potential noninvasive biomarkers of systemic immune changes during therapy [141]. Emerging observations such as atypical response patterns (e.g., pseudo-progression or pseudo-stability) further complicate clinical assessment and require careful radiologic and clinical correlation [142].

Predictive biomarker development remains an active area of investigation. While PD-L1 expression enriches for response in atezolizumab monotherapy, clinical benefit has also been observed in PD-L1–low or negative tumors, particularly in combination regimens [143]. Variability in PD-L1 assays and interpretation remains a major limitation in clinical practice [144,145]. Additional biomarkers, including treatment response kinetics and depth of response, are being explored as potential surrogate endpoints for survival [146]. Real-world studies further support the comparative effectiveness of immunotherapy regimens and highlight heterogeneity in outcomes across patient populations [147,148]. Combination strategies incorporating chemotherapy and anti-angiogenic agents are supported by a strong biological rationale, including enhanced antigen release, improved immune priming, and modulation of the tumor microenvironment toward an immune-permissive state, while meta-analyses comparing PD-1/PD-L1 inhibitors with docetaxel in previously treated NSCLC demonstrate a class-wide survival advantage for ICIs, reinforcing the paradigm shift in lung cancer therapy [149,150].

Economic considerations are increasingly relevant with the expanding use of immunotherapy. Cost-effectiveness analyses suggest that ICIs provide clinical benefit but are associated with substantial financial burden, particularly in earlier-stage and adjuvant settings [151,152,153]. These findings underscore the importance of value-based treatment strategies and healthcare resource optimization.

Taken together, atezolizumab has become an integral component of NSCLC management across multiple disease stages and treatment settings. Its clinical development illustrates the successful translation of immune checkpoint biology into durable clinical benefit while also highlighting the importance of careful interpretation of trial data in light of study design limitations, biomarker selection, and patient heterogeneity. Emerging approaches, including engineered cellular therapies designed to overcome PD-L1–mediated immunosuppression, may further expand the immunotherapeutic landscape in lung cancer [154].

Table 3 summarizes treatment-emergent adverse events associated with atezolizumab along with recommended management strategies, while Table 4 presents the major pivotal clinical trials evaluating atezolizumab in NSCLC.

## 4. Amivantamab

**Figure 4:**
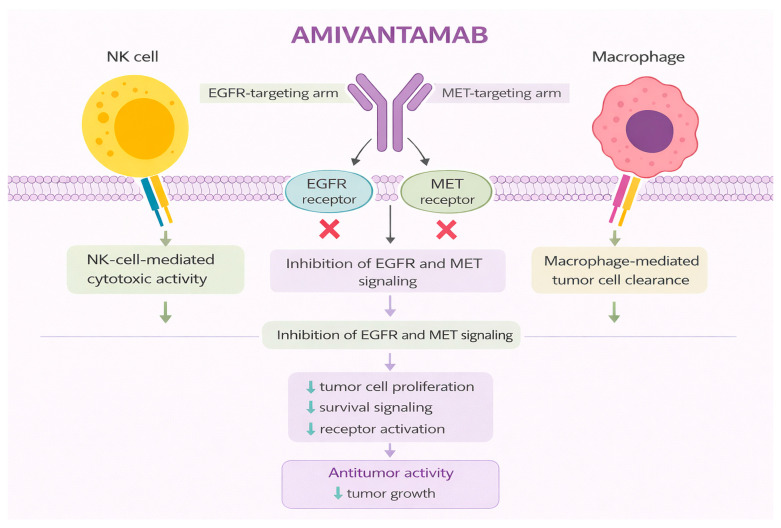
Mechanism of action of amivantamab. Original schematic illustration created by the authors based on the mechanism described in [178]. Amivantamab is a low-fucosylated bispecific IgG1 antibody targeting EGFR and c-MET that promotes Fc-independent receptor heterodimerization, internalization, and downregulation of oncogenic signaling while simultaneously inducing Fc-dependent immune effector mechanisms, including NK cell–mediated cytotoxicity (ADCC) and macrophage trogocytosis, to enhance tumor cell elimination. All abbreviations employed are defined in the text in the Abbreviations section.

Amivantamab is a fully human immunoglobulin G1 (IgG1) bispecific monoclonal antibody that simultaneously targets the extracellular domains of EGFR and MET, two key oncogenic drivers implicated in the pathogenesis and therapeutic resistance of NSCLC [156,157,158,159,160]. Unlike small-molecule tyrosine kinase inhibitors, amivantamab exerts its antitumor activity through multiple complementary mechanisms, including inhibition of ligand binding, receptor degradation, and immune effector–mediated cytotoxicity [159,161,162]. This dual-targeting strategy was specifically designed to address tumors harboring EGFR exon 20 insertion mutations, which are intrinsically resistant to first- and second-generation EGFR tyrosine kinase inhibitors and associated with poor clinical outcomes [163,164,165,166]. Figure 4 illustrates the mechanism of action of amivantamab.

EGFR exon 20 insertion mutations account for approximately 4–12% of all EGFR-mutant NSCLC cases and represent a molecularly heterogeneous subgroup characterized by steric hindrance within the ATP-binding pocket of the receptor, limiting the efficacy of conventional EGFR inhibitors [165,167,168]. Additionally, MET signaling has been implicated in both primary and acquired resistance to EGFR-targeted therapies, providing a strong biological rationale for dual EGFR–MET inhibition [160,169,170]. Preclinical studies demonstrated that amivantamab effectively induces receptor internalization and degradation while engaging immune effector cells through antibody-dependent cellular cytotoxicity and trogocytosis, resulting in sustained suppression of oncogenic signaling [171,172,173].

The clinical development of amivantamab progressed rapidly following encouraging preclinical data demonstrating favorable pharmacokinetics, sustained receptor occupancy, and a manageable safety profile, which supported further evaluation in molecularly selected NSCLC populations [50,159]. Early clinical evidence was generated in the phase I CHRYSALIS trial, a multicenter, open-label study evaluating amivantamab in patients with advanced or metastatic NSCLC harboring EGFR exon 20 insertion mutations who had progressed after platinum-based chemotherapy [50,51]. In this pivotal cohort, amivantamab demonstrated clinically meaningful antitumor activity, with an objective response rate (ORR) of approximately 40%, a median duration of response (DoR) of 11.1 months, and a median progression-free survival (PFS) of 8.3 months [160,174,175,176]. Responses were observed across a broad spectrum of EGFR exon 20 insertion variants, highlighting the activity of this agent in a genetically heterogeneous subgroup and supporting its regulatory approval for platinum-pretreated NSCLC with EGFR exon 20 insertion mutations [54,177]. However, interpretation of CHRYSALIS should consider its single-arm, open-label design, which limits comparative assessment and may introduce selection bias, particularly in a biomarker-defined population with limited therapeutic options.

Subsequent phase III trials have expanded the therapeutic role of amivantamab in earlier lines of therapy and additional EGFR-mutated populations. The PAPILLON study evaluated amivantamab combined with carboplatin and pemetrexed as first-line treatment for advanced NSCLC with EGFR exon 20 insertion mutations and demonstrated a significant improvement in PFS compared with chemotherapy alone (median PFS 11.4 vs. 6.7 months; HR 0.40), accompanied by a higher ORR (73% vs. 47%) and encouraging interim overall survival results [174,179,180]. While these findings support a shift toward earlier use, the interpretation of interim survival data requires caution pending mature overall survival outcomes.

In patients with common EGFR mutations (exon 19 deletion or L858R), the phase III MARIPOSA trial showed that the combination of amivantamab and the third-generation EGFR tyrosine kinase inhibitor lazertinib significantly prolonged PFS compared with osimertinib in previously untreated locally advanced or metastatic NSCLC (median PFS 23.7 vs. 16.6 months; HR 0.70), while maintaining high response rates in both treatment groups [181,182,183,184]. These findings support the concept that combining targeted antibodies with EGFR tyrosine kinase inhibitors may provide more comprehensive pathway inhibition than either modality alone [185,186,187,188,189]. However, cross-trial comparisons with established standards should be interpreted cautiously given differences in patient populations, study design, and treatment sequencing.

The therapeutic potential of amivantamab has also been investigated in patients with disease progression following prior targeted therapy. The phase III MARIPOSA-2 trial evaluated amivantamab-based combinations in patients with EGFR-mutated NSCLC progressing after osimertinib and demonstrated improved PFS with both amivantamab plus chemotherapy and amivantamab plus lazertinib combined with chemotherapy compared with chemotherapy alone (median PFS 6.3 and 8.3 vs. 4.2 months; HR 0.48 and 0.44, respectively), with substantially higher response rates [190,191]. In addition, the CHRYSALIS-2 study (Cohort A) assessed the combination of amivantamab and lazertinib in patients with EGFR exon 19 deletion or L858R mutations whose disease had progressed after osimertinib and platinum-based chemotherapy, reporting ORRs of 28% by investigator assessment and 35% by blinded independent central review, with a median DoR of 8.3 months and a median overall survival of 14.8 months [185,192,193]. Notably, CHRYSALIS-2 remains a non-randomized study, and its findings should therefore be interpreted as supportive but not definitive comparative evidence.

Overall, these studies highlight the expanding clinical role of amivantamab across multiple therapeutic settings in EGFR-mutated NSCLC, including first-line treatment, combination strategies with tyrosine kinase inhibitors, and post-resistance therapeutic approaches. However, heterogeneity in prior treatment exposure, molecular subgroups, and study design across trials complicates direct comparisons and underscores the need for continued randomized evidence.

The safety profile of amivantamab reflects its mechanism of action and target expression in normal tissues. The most common adverse events include infusion-related reactions, dermatologic toxicities such as rash and paronychia, and gastrointestinal symptoms, most of which occur early in treatment and are manageable with supportive care and dose modifications [174,194,195,196]. Infusion-related reactions are particularly frequent during the first administration, necessitating premedication and careful monitoring, but rarely lead to treatment discontinuation [197,198]. Overall, the safety profile has been considered acceptable in light of the clinical benefit observed in a population with limited therapeutic options.

Biomarker-driven patient selection is central to the clinical use of amivantamab. Its approved indication is restricted to NSCLC harboring EGFR exon 20 insertion mutations, underscoring the importance of comprehensive molecular testing using next-generation sequencing platforms [165,199,200]. Ongoing research aims to refine biomarker strategies further, including the identification of co-occurring alterations that may influence response or resistance, such as MET amplification or secondary EGFR mutations [166,201]. Nevertheless, reliance on specific molecular subsets may limit applicability to broader NSCLC populations and highlights the need for expanded biomarker frameworks.

In the rapidly evolving therapeutic landscape of NSCLC, amivantamab represents a significant advance in precision oncology, addressing a long-standing unmet need for patients with EGFR exon 20 insertion mutations. Its development highlights the potential of bispecific antibody engineering to overcome intrinsic resistance mechanisms and expand the scope of targeted therapy beyond traditional kinase inhibition [159,202]. As clinical experience grows and combination strategies mature, amivantamab is likely to play an increasingly important role in molecularly defined NSCLC, although ongoing studies are required to better define optimal sequencing, long-term outcomes, and comparative effectiveness [179,182,203].

Table 5 summarizes treatment-emergent adverse events associated with amivantamab and their management strategies, while Table 6 outlines the major pivotal clinical trials evaluating amivantamab in NSCLC.

## 5. Emerging Directions and Translational Insights in NSCLC

The future management of NSCLC is increasingly shaped by the convergence of molecular oncology, advanced antibody engineering, and integrated clinical decision-making. An expanding understanding of tumor biology—including signaling redundancy, immune evasion, and tumor–microenvironment interactions—has enabled the development of antibody-based targeted therapies that extend beyond classical kinase inhibition [208,209,210,211,212]. Agents such as ramucirumab, atezolizumab, and amivantamab exemplify complementary therapeutic strategies targeting angiogenesis (VEGFR-2), immune checkpoints (PD-L1), and oncogenic signaling (EGFR/MET), respectively. Their clinical success reflects a broader transition toward precision oncology paradigms that integrate tumor-intrinsic alterations with extrinsic processes such as angiogenesis, immune modulation, and adaptive resistance mechanisms [213,214].

A central challenge remains the refinement of biomarker-driven patient selection. While biomarker-based approaches are well established for amivantamab in EGFR exon 20 insertion–mutated NSCLC and for atezolizumab in PD-L1–defined populations, predictive biomarkers for antiangiogenic therapies such as ramucirumab remain incompletely characterized [215,216,217,218,219]. Consequently, patient selection for ramucirumab continues to rely primarily on clinical characteristics and prior treatment exposure rather than validated molecular predictors [220]. Ongoing translational research exploring angiogenic gene signatures, VEGF pathway activity, immune infiltration patterns, tumor mutational burden, and circulating biomarkers—including plasma proteins and circulating tumor DNA (ctDNA)—may enable more refined patient stratification and dynamic treatment adaptation [221,222,223,224,225,226].

Combination strategies represent a key direction for improving outcomes and are strongly supported by biological rationale across VEGFR-2, PD-L1, and EGFR/MET pathways. Inhibition of angiogenic signaling may remodel the tumor microenvironment by normalizing vasculature, alleviating hypoxia, reducing immunosuppressive cytokine gradients, and enhancing immune cell trafficking, thereby potentiating the efficacy of immune checkpoint inhibitors such as atezolizumab [227,228,229,230]. Similarly, bispecific targeting with agents such as amivantamab enables simultaneous pathway inhibition and may overcome intrinsic resistance mechanisms associated with tumor heterogeneity and signaling redundancy [214,231,232]. Ongoing and future phase III trials evaluating combinations of antibody-based therapies with immunotherapy, chemotherapy, and tyrosine kinase inhibitors are expected to further refine treatment algorithms across disease settings and lines of therapy [233,234,235,236,237,238].

Treatment sequencing and resistance management are becoming increasingly complex as patients are exposed to multiple therapeutic classes. Mechanisms of resistance include compensatory pathway activation, clonal evolution, intratumoral heterogeneity, and microenvironment-mediated immune escape [239,240,241,242]. In this context, an increasingly important component of treatment sequencing is the integration of longitudinal molecular monitoring, particularly through ctDNA analysis. Liquid biopsy provides a minimally invasive and dynamic approach to capturing tumor genomic evolution, enabling real-time assessment of resistance mechanisms that emerge during targeted therapy, especially in EGFR- and MET-driven disease settings.

ctDNA profiling has demonstrated clinical utility in detecting emergent MET amplification, a key bypass mechanism of resistance following EGFR tyrosine kinase inhibitor therapy, including osimertinib, as well as in identifying secondary EGFR resistance mutations affecting the kinase domain or extracellular regions, which may influence sensitivity to subsequent therapies such as amivantamab. This approach enables non-invasive, serial monitoring and offers advantages over conventional tissue biopsy, particularly in patients with limited tumor accessibility. Importantly, ctDNA-informed monitoring supports dynamic treatment adaptation across multiple lines of therapy. Following progression on osimertinib, ctDNA can guide the identification of actionable resistance mechanisms—such as MET amplification—that may inform the initiation of combination or bispecific targeting strategies. Similarly, during or after amivantamab-based therapy, serial ctDNA analysis may facilitate early detection of emerging resistance alterations, enabling timely therapeutic adjustments and potentially improving clinical outcomes. However, limitations remain, including reduced sensitivity in low-shedding tumors and challenges in distinguishing true clonal drivers from subclonal alterations or background noise. Despite these constraints, ctDNA represents a critical tool in advancing adaptive, biomarker-informed treatment strategies [217,223,243,244].

From a clinical trial perspective, innovative study designs—including basket trials, adaptive platform studies, and biomarker-enriched approaches—are essential to address molecular heterogeneity and accelerate drug development, particularly in rare molecular subgroups such as EGFR exon 20 insertion–mutated NSCLC [245,246,247,248]. The incorporation of translational endpoints, patient-reported outcomes, and real-world evidence will further enhance the interpretability and generalizability of trial results [249,250].

As antibody-based therapies are increasingly integrated into clinical practice, considerations related to toxicity management and healthcare resource utilization remain critical. Although the safety profiles of ramucirumab, atezolizumab, and amivantamab are generally predictable and manageable, proactive monitoring and multidisciplinary care are essential, particularly in the context of combination regimens [251,252,253].

In parallel, the growing economic burden associated with combination therapeutic strategies has emerged as a significant challenge. Regimens such as atezolizumab combined with bevacizumab, carboplatin, and paclitaxel (ABCP), as well as targeted combinations including amivantamab plus lazertinib, have demonstrated meaningful clinical benefit but are associated with substantial costs that may limit long-term sustainability within healthcare systems [225,254].

These economic challenges are further compounded by variability in global access and reimbursement frameworks, contributing to disparities in treatment availability across regions. In this context, cost-effectiveness analyses are increasingly important to inform evidence-based clinical and policy decision-making. Biomarker-driven patient selection represents a key strategy to optimize the value of high-cost therapies by identifying patients most likely to benefit, thereby improving both clinical outcomes and resource allocation efficiency. Furthermore, implementation of these regimens should be adapted to the specific context of individual healthcare systems, taking into account infrastructure, reimbursement policies, and resource availability [151,152,153,254].

In the broader context of oncology practice, effective implementation of antibody-based therapies requires multidisciplinary collaboration and equitable access to molecular diagnostics and targeted treatments [212,237,255,256]. Future advances in NSCLC will be driven by rational combination strategies, improved biomarker selection, and optimized treatment sequencing centered on VEGFR-2, PD-L1, and EGFR/MET pathway targeting. These developments are expected to enhance the precision and durability of therapeutic responses while maintaining a clear focus on clinically actionable, mechanism-based treatment strategies [257,258,259,260,261,262,263,264,265,266,267].

**Figure 5:**
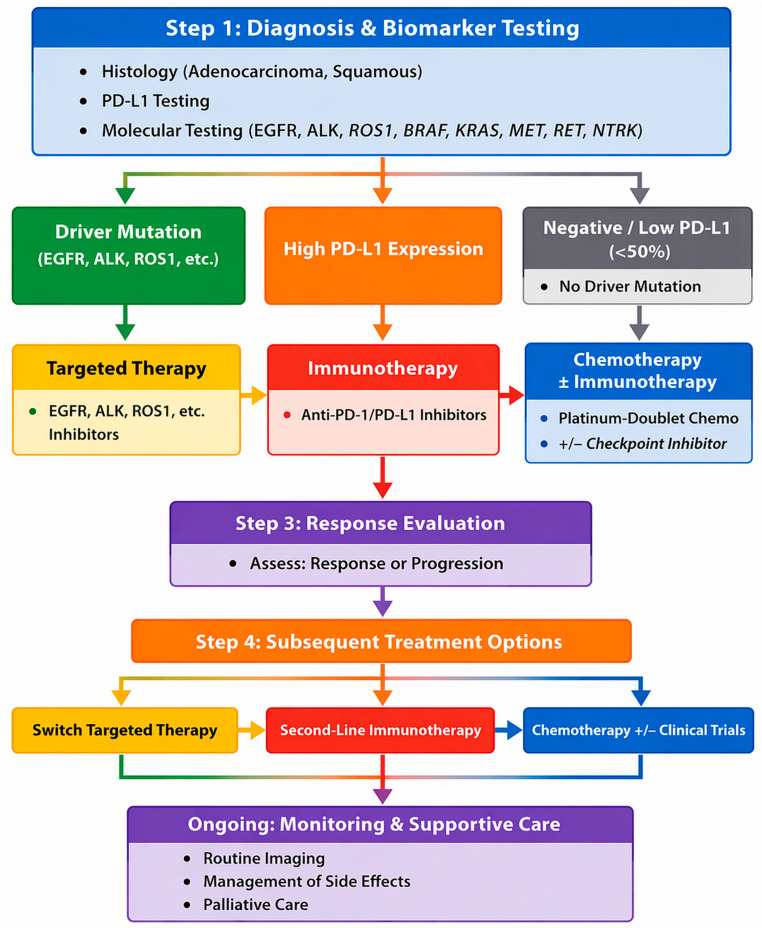
Therapeutic pathways and treatment algorithms in NSCLC integrating biomarkers and treatment sequencing. The figure depicts a stepwise NSCLC management strategy, starting with histologic and comprehensive biomarker assessment (PD-L1, EGFR, ALK, ROS1, BRAF, KRAS, MET, RET, NTRK), guiding treatment with targeted therapies for actionable mutations, first-line immunotherapy for high PD-L1 without drivers, and chemotherapy ± immunotherapy for low/negative PD-L1, followed by response-based adjustments, continuous monitoring, toxicity management, imaging, and integration of supportive and palliative care throughout the disease course. The presented algorithm represents an interpretative synthesis of current clinical evidence and pivotal trial data, aligned with key principles of NCCN and ESMO guidelines, including biomarker-driven treatment selection and therapy sequencing based on prior exposure and resistance mechanisms. It constitutes an original graphical representation and does not reproduce or replicate any specific published guideline or source. Areas reflecting emerging or investigational strategies are indicated and should be interpreted within the context of evolving clinical evidence. All abbreviations employed are defined in the text in the Abbreviations section.

Table 7 summarizes current management strategies in NSCLC, while Figure 5 presents a biomarker-driven treatment algorithm that integrates molecular profiling with clinical staging to guide therapeutic sequencing, providing a structured, clinically oriented synthesis of the current therapeutic landscape in NSCLC.

## 6. Conclusions

The therapeutic landscape of NSCLC has undergone a profound transformation with the integration of antibody-based targeted therapies, reflecting broader advances in molecular oncology, immune biology, and precision medicine. Ramucirumab, atezolizumab, and amivantamab represent distinct yet complementary examples of how rationally engineered monoclonal and bispecific antibodies can effectively target critical biological processes involved in tumor progression, immune evasion, angiogenesis, and therapeutic resistance. Collectively, these agents have expanded treatment options for patient populations historically associated with limited benefit from conventional cytotoxic chemotherapy, including those with angiogenesis-driven disease, immune-responsive tumors, and rare but clinically challenging molecular subtypes.

Robust clinical evidence from pivotal trials has established ramucirumab as an effective antiangiogenic therapy in previously treated advanced NSCLC, atezolizumab as a cornerstone immunotherapy across multiple disease stages and treatment settings, and amivantamab as a landmark targeted agent for patients with EGFR exon 20 insertion–mutated NSCLC, a subgroup characterized by intrinsic resistance to most EGFR tyrosine kinase inhibitors. These advances underscore the growing importance of integrating molecular, immunologic, histologic, and clinical factors into individualized treatment strategies and highlight the necessity of moving beyond uniform treatment paradigms in a biologically heterogeneous disease.

Despite these successes, several challenges remain. Predictive biomarkers for antibody-based therapies—particularly for antiangiogenic agents such as ramucirumab—remain incompletely defined, limiting optimal patient selection and treatment personalization. Although biomarkers such as PD-L1 expression and specific genomic alterations have improved selection for immune checkpoint inhibition and bispecific antibody therapy, resistance mechanisms—both intrinsic and acquired—continue to constrain the durability of clinical benefit. These challenges emphasize the need for deeper mechanistic understanding, longitudinal molecular monitoring, and adaptive therapeutic strategies capable of addressing tumor evolution over time.

Looking ahead, continued advances in antibody engineering, including bispecific and multispecific constructs, offer substantial promise for overcoming resistance pathways and enhancing therapeutic efficacy. Rational combination strategies integrating antibody-based therapies with immunotherapy, targeted small-molecule inhibitors, chemotherapy, and biomarker-guided sequencing approaches are likely to further redefine treatment algorithms and expand the role of antibodies earlier in the disease course. In parallel, innovative clinical trial designs, real-world evidence generation, and health economic analyses will be essential to ensure that these advances translate into meaningful, sustainable, and equitable clinical benefit.

In summary, ramucirumab, atezolizumab, and amivantamab exemplify the expanding and evolving role of antibody-based targeted therapies in NSCLC. Their development and clinical implementation illustrate the progress achieved through biologically informed drug design and precision oncology frameworks. Continued efforts in biomarker discovery, rational combination development, and optimized clinical integration are expected to further refine their therapeutic impact, with the ultimate goal of improving survival, preserving quality of life, and moving closer to durable disease control for patients with advanced NSCLC.

## Figures and Tables

**Table 1:** TEAEs and management strategies for ramucirumab according to [38,104]. All abbreviations employed are defined in the text in the Abbreviations section.

TEAE	Frequency/ Severity	Timing/ Clinical Features	Recommended Management
Hematologic toxicity (neutropenia, febrile neutropenia, thrombocytopenia)	Neutropenia: all-grade 55%, Grade 3–4 49%; febrile neutropenia: 16% (Grade 3–4 16%); thrombocytopenia: all-grade 13%, Grade 3–4 3% (REVEL)	Typically chemotherapy-related; ANC nadir ~7–10 days post-docetaxel; infection or bleeding risk	CBC monitoring; manage per institutional neutropenia/febrile neutropenia pathways; empiric antibiotics if febrile; consider G-CSF; transfusion/supportive care as indicated
Fatigue/asthenia	All-grade 55%; Grade 3–4 14% (REVEL)	Often cumulative; multifactorial (anemia, infection, sleep, nutrition)	Assess reversible causes; supportive care, activity pacing, sleep optimization
Mucositis (stomatitis/mucosal inflammation)	All-grade 37%; Grade 3–4 7% (REVEL)	Post-chemotherapy; oral pain, ulcers, reduced intake	Oral hygiene, topical analgesics; hydration/nutrition; follow mucositis guidelines; consider dose delays if severe
Hypertension (VEGFR2 class effect)	All-grade 11%; Grade 3–4 6% (REVEL)	May occur early or later; often asymptomatic	Optimize BP prior to therapy; monitor regularly; withhold treatment for severe hypertension; discontinue if uncontrolled or crisis occurs
Bleeding events (epistaxis, pulmonary hemorrhage/hemoptysis)	Epistaxis: all-grade 19%, Grade 3–4 < 1%; pulmonary hemorrhage: ~7–10% overall, Grade ≥ 3 ~1–2% (REVEL)	Usually mild mucosal bleeding; hemoptysis may indicate serious complication	Local measures for minor bleeding; review concomitant drugs; urgent evaluation for significant bleeding; discontinue treatment in severe cases
Proteinuria (including nephrotic syndrome)	~3.3% clinically relevant ADRs (REVEL)	May be cumulative; asymptomatic or edema/foamy urine	Monitor urine (dipstick/UPCR); dose modification or discontinuation based on protein levels
Infusion-related reactions	Uncommon; may be severe	Typically during first/second infusion; hypersensitivity-type symptoms	Premedication and monitoring; reduce infusion rate for mild reactions; discontinue permanently for severe reactions
Serious vascular and organ complications (arterial thromboembolism, GI perforation, impaired wound healing, PRES)	Rare to very rare but potentially life-threatening	Acute neurologic/cardiac symptoms; abdominal pain; surgical complications; neurologic signs (PRES)	Urgent evaluation; permanent discontinuation in severe cases; perioperative treatment interruption as appropriate

**Table 2:** Major pivotal clinical trials of ramucirumab. All abbreviations employed are defined in the text in the Abbreviations section.

Trial	Population/ Cancer Setting	Design/ Combination	Key Findings	Inclusion/ Eligibility Criteria
REVEL [38]	Stage IV NSCLC; progression after platinum-based chemotherapy (second-line); squamous and non-squamous histology	Phase III, randomized, double-blind; ramucirumab + docetaxel vs. placebo + docetaxel	Improved OS (10.5 vs. 9.1 months); improved PFS (4.5 vs. 3.0 months); benefit across histologies	ECOG 0–1; prior platinum-based chemotherapy; adequate organ function; excluded patients with significant hemoptysis or uncontrolled hypertension
RELAY [81]	Metastatic EGFR-mutated (exon 19 deletion or L858R) NSCLC; first-line	Phase III, randomized, double-blind; ramucirumab + erlotinib vs. placebo + erlotinib	Improved PFS (19.4 vs. 12.4 months); delayed disease progression; manageable safety profile	Treatment-naïve metastatic EGFR exon 19 deletion or L858R mutation; ECOG 0–1; no T790M mutation; adequate organ function
Phase II Japanese study (JVBT) [78]	Previously treated advanced NSCLC (Japanese population)	Phase II, single-arm; ramucirumab + docetaxel	Demonstrated clinical activity and tolerable safety supporting phase III development	Prior platinum-based chemotherapy; ECOG 0–1; measurable disease per RECIST; adequate hematologic and organ function
PLEURAM study [79,80]	Previously platinum-treated NSCLC with malignant pleural effusion	Phase II, single-arm; ramucirumab + docetaxel	Demonstrated antitumor activity in patients with malignant pleural effusion; manageable safety profile; supported feasibility of combination in this high-risk subgroup	Prior platinum-based chemotherapy; presence of malignant pleural effusion; ECOG 0–1; measurable disease; adequate hematologic and organ function
Phase II docetaxel + ramucirumab + pembrolizumab study [94]	Metastatic or recurrent NSCLC progressing after platinum-doublet chemotherapy and prior PD-1/PD-L1 inhibitor	Phase II, single-arm; docetaxel + ramucirumab + pembrolizumab	Demonstrated encouraging antitumor activity and manageable safety in patients previously treated with chemotherapy and ICIs	Prior platinum-doublet chemotherapy and PD-1/PD-L1 blockade; metastatic or recurrent NSCLC; ECOG 0–1; adequate organ function
Lung-MAP S1800A [95]	Advanced NSCLC previously treated with immunotherapy	Randomized phase II trial; ramucirumab + docetaxel	Improved OS compared with standard of care (median OS 14.5 vs. 11.6 months); manageable safety; supported VEGFR2 + PD-1 strategy after ICI progression	Prior platinum-based chemotherapy and ICI; advanced NSCLC; ECOG 0–1; measurable disease; adequate organ function

**Table 3:** TEAEs and management strategies for atezolizumab according to [44,113,140,155]. All abbreviations employed are defined in the text in the Abbreviations section.

TEAE	Frequency/ Severity	Timing/ Clinical Features	Recommended Management
General and constitutional symptoms (fatigue, arthralgia/myalgia)	Fatigue ~15–25%; arthralgia/myalgia ~5–10%; mostly Grade 1–2	Can occur anytime; nonspecific fatigue, musculoskeletal discomfort	Exclude endocrine causes (TSH, cortisol); supportive care; NSAIDs for mild pain; consider low-dose corticosteroids if persistent; hold therapy for Grade ≥ 3
Dermatologic toxicity (rash, pruritus)	Common (~10–20%); usually Grade 1–2	Early onset (weeks); maculopapular rash, itching	Topical corticosteroids, antihistamines; continue therapy for Grade 1–2; hold and initiate systemic steroids (0.5–1 mg/kg/day prednisone) for Grade ≥ 3
Pulmonary toxicity (immune-related pneumonitis)	~3–5%; Grade 3–4 ~1–2%	Median onset 2–6 months; cough, dyspnea, CT changes	Grade 1: monitor; Grade 2: hold therapy, prednisone 1 mg/kg/day; Grade ≥ 3: permanently discontinue; IV steroids ± additional immunosuppression
Gastrointestinal toxicity (immune-mediated colitis/diarrhea)	~5–10%; severe <2%	Typically within 6–8 weeks; diarrhea, abdominal pain	Grade 1: symptomatic care; Grade 2: hold therapy, prednisone 1 mg/kg/day; Grade ≥ 3: discontinue; IV steroids ± infliximab if refractory
Hepatic toxicity (immune-mediated hepatitis)	~2–6%; Grade 3–4 <2%	Often asymptomatic; detected on labs (↑AST/ALT)	Grade 2: hold therapy, prednisone 0.5–1 mg/kg/day; Grade ≥ 3: discontinue; IV steroids; consider mycophenolate if refractory
Endocrine immune-related events (thyroid dysfunction, hypophysitis, adrenal insufficiency)	Thyroid ~8–15%; others rare (<1–2%)	Variable onset; fatigue, weight changes, hypotension, electrolyte abnormalities	Routine endocrine monitoring; hormone replacement as needed; corticosteroids for acute adrenal insufficiency
Infusion-related reactions	<5%; usually mild	During or shortly after infusion; fever, chills	Interrupt/slow infusion; antihistamines/antipyretics; discontinue if severe

**Table 4:** Major pivotal clinical trials of atezolizumab. All abbreviations employed are defined in the text in the Abbreviations section.

Trial	Population/ Cancer Setting	Design/ Combination	Key Findings	Inclusion/ Eligibility Criteria
POPLAR [113]	Previously treated (≥2 L) advanced/metastatic NSCLC	Phase II, randomized: atezolizumab vs. docetaxel	Improved OS vs. docetaxel (median 12.6 vs. 9.7 months; HR 0.73)	Prior platinum-based chemotherapy; stage IIIB/IV NSCLC; measurable disease (RECIST); ECOG PS 0–1
OAK [44]	Previously treated advanced/metastatic NSCLC	Phase III, randomized: atezolizumab vs. docetaxel	Improved OS in ITT population (13.8 vs. 9.6 months; HR 0.73); benefit across PD-L1 subgroups	Prior platinum chemotherapy; stage IIIB/IV; ECOG PS 0–1; all PD-L1 expression levels eligible
IMpower110 [119]	First-line metastatic NSCLC (squamous and nonsquamous), PD-L1–selected	Phase III, randomized: atezolizumab monotherapy vs. platinum chemotherapy	In PD-L1–high EGFR/ALK wild-type subgroup: OS 20.2 vs. 13.1 months; HR 0.59	No prior systemic therapy for metastatic disease; PD-L1 expression ≥1% (SP142 assay); EGFR/ALK wild-type in primary analysis population; ECOG PS 0–1
IMpower150 [45,123]	First-line metastatic nonsquamous NSCLC	Phase III, randomized: ABCP (atezolizumab + bevacizumab + carboplatin + paclitaxel) vs. BCP	Improved OS (19.5 vs. 14.7 months; HR 0.80 in WT population); improved PFS	Untreated stage IV nonsquamous NSCLC; ECOG PS 0–1; EGFR/ALK alterations allowed after TKI failure (exploratory subgroup)
IMpower130 [46]	First-line metastatic nonsquamous NSCLC	Phase III, randomized: atezolizumab + carboplatin + nab-paclitaxel vs. chemotherapy	OS improved (18.6 vs. 13.9 months; HR 0.79); PFS 7.0 vs. 5.5 months; HR 0.64	Stage IV nonsquamous NSCLC; no prior systemic therapy for metastatic disease; EGFR/ALK wild-type in primary analysis; ECOG PS 0–1
IMpower131 [126]	First-line metastatic squamous NSCLC	Phase III, randomized: atezolizumab + carboplatin + nab-paclitaxel vs. chemotherapy	PFS improved (6.3 vs. 5.6 months; HR 0.71); OS not statistically significant overall	Stage IV squamous NSCLC; treatment-naïve; ECOG PS 0–1; broad PD-L1 inclusion
IMpower010 [47]	Adjuvant setting after complete resection and platinum chemotherapy (stage IB–IIIA)	Phase III, randomized: atezolizumab (1 year) vs. best supportive care	DFS benefit in stage II–IIIA with PD-L1 ≥1% (HR 0.66); DFS improved in stage II–IIIA overall (HR 0.79)	Completely resected stage IB (≥4 cm)–IIIA NSCLC; 1–4 cycles adjuvant platinum chemotherapy; ECOG PS 0–1

**Table 5:** TEAEs and management strategies for amivantamab according to [50,55,204,205,206,207]. All abbreviations employed are defined in the text in the Abbreviations section.

TEAE	Frequency/ Severity	Timing/ Clinical Features	Recommended Management
Infusion-related reactions (IRR)	Very common ~66–67%; Grade ≥ 3 in a minority	Predominantly during first infusion (Cycle 1 Day 1); chills, dyspnea, flushing, nausea, hypotension, bronchospasm	Premedication; split first dose over 2 days; interrupt infusion at onset of symptoms and resume at reduced rate once resolved; discontinue permanently for life-threatening reactions
Dermatologic toxicity (rash, paronychia/nail toxicity)	Rash ~76–86%; paronychia ~45–47%; Grade ≥ 3 less common	Rash typically early (weeks); paronychia develops later (weeks–months); inflammatory skin and periungual changes	Topical corticosteroids, antihistamines; oral antibiotics (e.g., doxycycline) for acneiform rash; nail care and antiseptics; systemic steroids and treatment interruption for severe cases; dermatology referral as needed
Mucosal toxicity (stomatitis/mucositis)	~21–24%	Oral soreness, ulcers, dysgeusia; may affect intake	Oral hygiene, saline/bicarbonate rinses, topical anesthetics; consider topical steroids; hold treatment for severe cases; ensure nutritional support
Edema and metabolic effects (peripheral edema, hypoalbuminemia)	Edema ~26%; hypoalbuminemia ~31%	Peripheral swelling; laboratory-detected hypoalbuminemia contributing to edema/fatigue	Evaluate underlying causes (cardiac, renal, hepatic, nutritional); supportive measures (compression, elevation); diuretics if appropriate; monitor labs; consider treatment interruption if severe
Thromboembolic events (VTE)	Reported; potentially serious	Variable timing; symptoms include limb swelling, dyspnea, chest pain	Prompt diagnostic evaluation; initiate anticoagulation unless contraindicated; consider treatment interruption based on severity
Pulmonary toxicity (interstitial lung disease/pneumonitis)	Uncommon but potentially serious	New/worsening cough, dyspnea, hypoxia; imaging abnormalities	Hold treatment; perform diagnostic workup; initiate corticosteroids if drug-related; permanently discontinue in severe cases

**Table 6:** Major pivotal clinical trials of amivantamab. All abbreviations employed are defined in the text in the Abbreviations section.

Trial	Population/ Cancer Setting	Design/ Combination	Key Findings	Inclusion/ Eligibility Criteria
CHRYSALIS (EGFR Exon20ins cohort) [50]	Advanced/metastatic NSCLC with EGFR exon 20 insertion, post-platinum	Phase I (dose-expansion); amivantamab monotherapy	ORR 40%; median DoR 11.1 mo; median PFS 8.3 mo (pivotal efficacy population)	EGFR Exon20ins NSCLC; progressed after platinum chemotherapy; treated at RP2D; measurable disease per RECIST; typical ECOG PS 0–1
PAPILLON [174]	1 L advanced NSCLC with EGFR exon 20 insertion	Phase III, randomized; amivantamab + carboplatin/pemetrexed vs. chemo	Median PFS 11.4 vs. 6.7 mo; HR 0.40; ORR 73% vs. 47% (BICR). Interim OS HR 0.67 (not significant at interim)	Treatment-naïve advanced NSCLC with EGFR Exon20ins; randomized 1:1; primary endpoint PFS by BICR
MARIPOSA [181]	1 L locally advanced/metastatic NSCLC with EGFR exon 19 del or L858R	Phase III, randomized (2:2:1); amivantamab + lazertinib vs. osimertinib vs. lazertinib	Median PFS 23.7 vs. 16.6 mo; HR 0.70; ORR 86% vs. 85%; interim OS HR 0.80	Previously untreated; EGFR exon 19 deletion or L858R; locally advanced/metastatic; primary endpoint PFS by BICR
MARIPOSA-2 [190]	Post-osimertinib EGFR-mutant advanced/metastatic NSCLC (ex19del/L858R)	Phase III, randomized (2:2:1); amivantamab-chemo and amivantamab-lazertinib-chemo vs. chemo	Median PFS 6.3 (amivantamab-chemo) and 8.3 (amivantamab-lazertinib-chemo) vs. 4.2 mo; HR 0.48 and 0.44; ORR 64% and 63% vs. 36%	EGFR ex19del/L858R; locally advanced/metastatic; progressed on osimertinib; randomized to 3 arms; dual primary endpoints PFS vs. chemo
CHRYSALIS-2 (Cohort A) [185]	EGFR ex19del/L858R advanced NSCLC after osimertinib + platinum	Phase I/1b cohort; amivantamab + lazertinib	ORR 28% (investigator)/35% (BICR); median DoR 8.3 mo; median PFS 4.5 mo; median OS 14.8 mo	EGFR ex19del or L858R; progressed on/after osimertinib and platinum chemotherapy; primary endpoint ORR

**Table 7:** Contemporary management of NSCLC: clinically focused overview of current treatment strategies, including indications, representative regimens, biomarkers, toxicity considerations, and supporting evidence. All abbreviations employed are defined in the text in the Abbreviations section.

Modality	Indication/ When Used	Example Regimens/ Agents	Key Evidence	Biomarkers/ Toxicity
Surgery (with nodal evaluation)	Resectable Stage I–II and selected IIIA	Lobectomy (preferred); segmentectomy (≤2 cm peripheral tumors); systematic lymph node dissection	LACE meta-analysis; NCCN Guidelines [268,269]	Molecular profiling recommended post-resection. Surgical risks: pneumonia, atrial fibrillation, air leak
Adjuvant Platinum Chemotherapy	Resected Stage II–IIIA; selected high-risk IB	Cisplatin + vinorelbine; cisplatin + pemetrexed (nonsquamous)	LACE meta-analysis [268]	Nephrotoxicity, neuropathy, cytopenias
Adjuvant EGFR TKI	Resected Stage IB–IIIA with EGFR exon 19 deletion or L858R mutation	Osimertinib	ADAURA (DFS and OS benefit) [270,271]	Requires EGFR mutation testing; diarrhea, rash, QT prolongation, ILD
Adjuvant Immunotherapy	Resected Stage II–IIIA (post-chemotherapy); PD-L1–directed use depending on agent	Atezolizumab; Pembrolizumab	IMpower010; KEYNOTE-091 [47,272]	PD-L1 testing recommended; immune-related adverse events (pneumonitis, endocrinopathies)
Neoadjuvant Chemo-Immunotherapy	Resectable Stage II–IIIA	Nivolumab + platinum doublet	CheckMate 816 [273]	Immune-related toxicities + chemotherapy-related cytopenias
Definitive Concurrent Chemoradiotherapy (cCRT)	Unresectable Stage III (curative intent)	Platinum doublet + thoracic radiotherapy	Standard of care; NCCN [269]	Esophagitis, radiation pneumonitis, cytopenias
Consolidation Immunotherapy (Post-cCRT)	Unresectable Stage III without progression after cCRT	Durvalumab	PACIFIC trial (OS benefit; 5-year outcomes) [274,275]	Increased pneumonitis risk after RT
Consolidation EGFR TKI (Stage III EGFR+)	EGFR-mutated Stage III after cCRT	Osimertinib	LAURA trial [276]	EGFR mutation required; ILD risk
First-Line Immunotherapy (Metastatic, PD-L1 ≥ 50%)	Advanced/metastatic NSCLC without actionable driver mutation	Pembrolizumab	KEYNOTE-024 [23]	PD-L1 TPS ≥50%; immune-related adverse events
First-Line Chemo-Immunotherapy	Metastatic NSCLC without actionable driver (all PD-L1 levels)	Pembrolizumab + platinum doublet (KEYNOTE-189 nonsquamous; KEYNOTE-407 squamous)	KEYNOTE-189; KEYNOTE-407 [277,278]	Combined immune and chemotherapy toxicity
EGFR-Targeted Therapy (Metastatic)	EGFR exon 19 deletion or L858R mutation	Osimertinib	FLAURA [279]	EGFR mutation required; QT prolongation, ILD
ALK-Targeted Therapy	ALK rearranged metastatic NSCLC	Alectinib; Lorlatinib	ALEX; CROWN [280,281]	CNS effects (lorlatinib), myalgia, liver enzyme elevation
KRAS G12C Inhibitors (Later Line)	Previously treated KRAS G12C-mutant NSCLC	Sotorasib; Adagrasib	CodeBreaK; KRYSTAL studies [282,283]	Hepatotoxicity, diarrhea, nausea
HER2-Directed Therapy	Previously treated HER2-mutant NSCLC	Trastuzumab deruxtecan	DESTINY-Lung02 [284]	ILD/pneumonitis risk; myelosuppression
Anti-VEGF + Chemo-Immunotherapy	Selected metastatic nonsquamous NSCLC	Atezolizumab + bevacizumab + carboplatin + paclitaxel	IMpower150 [45]	Hypertension, bleeding risk (bevacizumab), immune toxicity

## Data Availability

No new data were created or analyzed in this study. Data sharing is not applicable to this article.

## Abbreviations

The following abbreviations are used in this manuscript:

2L	Second Line
ABCP	Atezolizumab + Bevacizumab + Carboplatin + Paclitaxel
ACTH	Adrenocorticotropic Hormone
ADC	Antibody–Drug Conjugate
AE	Adverse Event
ALEX	Alectinib versus Crizotinib in Untreated ALK-Positive NSCLC Trial
ALK	Anaplastic Lymphoma Kinase
ALT	Alanine Aminotransferase
ANC	Absolute Neutrophil Count
anti-c-MET	Antibody Targeting c-MET
anti-EGFR	Antibody Targeting EGFR
AST	Aspartate Aminotransferase
ATE	Arterial Thromboembolic Event
BCP	Bevacizumab + Carboplatin + Paclitaxel
BEV	Bevacizumab
BICR	Blinded Independent Central Review
BP	Blood Pressure
BRAF	B-Raf Proto-Oncogene, Serine/Threonine Kinase
CBC	Complete Blood Count
cCRT	Concurrent Chemoradiotherapy
CHEMO	Chemotherapy
CNS	Central Nervous System
CT	Computed Tomography
CTCAE	Common Terminology Criteria for Adverse Events
CTL	Cytotoxic T Lymphocyte
DFS	Disease-Free Survival
DOR	Duration of Response
DVT	Deep Vein Thrombosis
ECOG	Eastern Cooperative Oncology Group
ECOG PS	Eastern Cooperative Oncology Group Performance Status
EFS	Event-Free Survival
EGFR	Epidermal Growth Factor Receptor
ex19del	Exon 19 Deletion
Exon20ins	Exon 20 Insertion
Fc	Fragment Crystallizable Region
FDA	Food and Drug Administration
G-CSF	Granulocyte Colony-Stimulating Factor
GI	Gastrointestinal
HER2	Human Epidermal Growth Factor Receptor 2
HR	Hazard Ratio
IB	Stage IB
ICI(s)	Immune Checkpoint Inhibitor(s)
ILD	Interstitial Lung Disease
IMpower010	Adjuvant Atezolizumab Trial
IMpower150	Atezolizumab plus Bevacizumab and Chemotherapy in Metastatic NSCLC Trial
IO	Immuno-Oncology
irAE	Immune-Related Adverse Event
IRR	Infusion-Related Reaction
ITT	Intention-To-Treat
IV	Intravenous
JVBT	Japanese Phase II Trial Code
KRAS	Kirsten Rat Sarcoma Viral Oncogene Homolog
L858R	Leucine-to-Arginine Substitution at Codon 858 (EGFR Mutation)
LACE	Lung Adjuvant Cisplatin Evaluation
LAURA	Osimertinib after Chemoradiotherapy in EGFR-Mutated Stage III NSCLC Trial
MEK	Mitogen-Activated Protein Kinase Kinase
MET	Mesenchymal–Epithelial Transition Factor
MHC	Major Histocompatibility Complex
MI	Myocardial Infarction
NCCN	National Comprehensive Cancer Network
NGS	Next-Generation Sequencing
NK cell	Natural Killer Cell
NSAIDs	Nonsteroidal Anti-Inflammatory Drugs
NSCLC	Non-Small Cell Lung Cancer
NTRK	Neurotrophic Tyrosine Receptor Kinase
ORR	Objective Response Rate
OS	Overall Survival
P	Phosphate Group (Phosphorylation)
PACIFIC	Durvalumab after Chemoradiotherapy in Stage III NSCLC Trial
PD-1	Programmed Cell Death Protein 1
PD-L1	Programmed Death-Ligand 1
PE	Pulmonary Embolism
PFS	Progression-Free Survival
PI3K	Phosphoinositide 3-Kinase
PKC	Protein Kinase C
PRES	Posterior Reversible Encephalopathy Syndrome
RAF	Rapidly Accelerated Fibrosarcoma Kinase
RAS	Rat Sarcoma Small GTPase
RECIST	Response Evaluation Criteria in Solid Tumors
RET	Rearranged during Transfection
REVEL	Randomized Phase III Clinical Trial of Ramucirumab Plus Docetaxel in Previously Treated NSCLC
ROS1	ROS Proto-Oncogene 1 Receptor Tyrosine Kinase
RP2D	Recommended Phase 2 Dose
RT	Radiotherapy
SBRT	Stereotactic Body Radiotherapy
TEAE	Treatment-Emergent Adverse Event
TCR	T Cell Receptor
TKI	Tyrosine Kinase Inhibitor
TPS	Tumor Proportion Score
TSH	Thyroid-Stimulating Hormone
T790M	Threonine-to-Methionine Substitution at Codon 790 (EGFR Resistance Mutation)
UPCR	Urine Protein-to-Creatinine Ratio
VEGFA	Vascular Endothelial Growth Factor A
VEGFR-2/VEGFR2	Vascular Endothelial Growth Factor Receptor 2
VTE	Venous Thromboembolism
WT	Wild Type
